# Pain in Children: Assessment and Nonpharmacological Management

**DOI:** 10.1155/2010/474838

**Published:** 2010-07-25

**Authors:** Rasha Srouji, Savithiri Ratnapalan, Suzan Schneeweiss

**Affiliations:** Division of Paediatric Emergency Medicine, Department of Paediatrics, The Hospital for Sick Children, University of Toronto, 555 University Avenue, Toronto, ON, Canada M5G 1X8

## Abstract

Pain perception in children is complex, and is often difficult to assess. In addition, pain management in children is not always optimized in various healthcare settings, including emergency departments. A review of pain assessment scales that can be used in children across all ages, and a discussion of the importance of pain in control and distraction techniques during painful procedures are presented. Age specific nonpharmacological interventions used to manage pain in children are most effective when adapted to the developmental level of the child. Distraction techniques are often provided by nurses, parents or child life specialists and help in pain alleviation during procedures.

## 1. Introduction

For pediatric patients presenting to the emergency department, medical procedures are often painful, unexpected, and heightened by situational stress and anxiety leading to an overall unpleasant experience. Although the principles of pain evaluation and management apply across the human lifespan, infants and children present unique challenges that necessitate consideration of the child's age, developmental level, cognitive and communication skills, previous pain experiences, and associated beliefs [1]. According to the International Association for the Study of Pain, “Pain is an unpleasant sensory and emotional experience associated with actual or potential tissue damage”. Perception of pain in pediatrics is complex, and entails physiological, psychological, behavioral, and developmental factors [1]. However, in spite of its frequency, pain in infants, children, and adolescent is often underestimated and under treated [2]. It has also been shown that infants and children, who experience pain in early life, show long-term changes in terms of pain perception and related behaviors [2]. Health care professionals in this setting have a responsibility to reduce pain and anxiety as much as possible while maintaining patient safety. 

Pain in infants and children can be difficult to assess which has led to the creation of numerous age-specific pain management tools and scores. Health care workers need to be able to detect the symptoms and signs of pain in different age groups and determine whether these symptoms are caused by pain or other factors [1]. It is difficult for health care professionals to foresee which measurement systems apply to accurately measure pain in the pediatric population [1]. Health care professionals often prefer practical methods, which reliably track the child's pain experience and pain control over time whereas researchers tend to focus on tools, which are meticulously proven for reliability with different observers. Thus a balance may be hard to achieve [1]. Barriers to pain management in children are numerous and include inaccuracies regarding pathophysiological mechanisms of pain with statements such as “children do not feel pain the way adults do” [3], fears regarding the use of pharmacological agents and deficits in knowledge of methods of pain assessment [3, 4]. These myths and other factors such as personal values and beliefs, prevent adequate identification and alleviation of pain for all children [2, 3].

Effective care in pediatrics requires special attention to the developmental stage of the child. Current research does not adequately discuss the effectiveness of certain tools and measurements used to assess pain in children at various ages [5]. The experience of pain and coping strategies from developmental perspective is also limited. In this paper, our aim is to address potential sources of pain measurement, and responses to pain control and distraction based on pediatric developmental stages. Pharmacological pain management will not be discussed, as it is beyond the intended scope of this article.

## 2. Pain Assessment Tools

Accurate pain measurements in children are difficult to achieve. Three main methods are currently used to measure pain intensity: self report, behavioral, and physiological measures. Self-report measures are optimal and the most valid [4]. Both verbal and nonverbal reports require a certain level of cognitive and language development for the child to understand and give reliable responses [4]. Children's capability to describe pain increases with age and experience, and changes throughout their developmental stages [4]. Although, observed reports of pain and distress provide helpful information, particularly for younger children, they are reliant on the individuals completing the report [6]. Behavioral measures consist of assessment of crying, facial expressions, body postures, and movements. They are more frequently used with neonates, infants, and younger children where communication is difficult [7]. Physiological measures include assessment of heart rate, blood pressure, respiration, oxygen saturation, palmer sweating, and sometimes neuro-endocrine responses [8]. They are however generally used in combination with behavioral and self-report measures, as they are usually valid for short duration acute pain and differ with the general health and maturational age of the infant or child [8]. In addition, similar physiological responses also occur during stress which results in difficulty distinguishing stress versus pain responses. A summary of the following pain assessment tools by age can be found in Table 1.

### 2.1. Neonates and Infants

Despite early studies, current research supports that infants possess the anatomical and functional requirements to perceive pain [9]. Recent studies also demonstrate that infants elicit certain behavioral responses to pain perception [10]. Pain in infants, despite this data, remains under-treated and often mismanaged [11]. The most common pain measures used for infants are behavioral. These measures include crying, facial expressions, body posture, and movements. The quality of these behaviors depends on the infant's gestational age, and maturity [12]. Preterm or acutely ill infants, for example, do not illicit similar responses to pain due to illness and lack of energy. In addition, interpretation of crying in infants is especially difficult as it may indicate general distress rather than pain. Cry characteristics are also not good indicators in preterm or acutely ill infants, as it is difficult for them to produce a robust cry [12].

Numerous scales are currently available to measure behavioral indicators in infants, the most common being the Neonatal Facial Coding System (NFCS) and the Neonatal Infant Pain Scale (NIPS). Other scales used with infants are composite measurement scales, meaning they use a combination of behavioral and physiological measures. Some scales also take into consideration gestational age and the general behavioral state of the infant [13]. Examples of these scales are The Premature Infant Pain Profile (PIPP), Crying Requires Increased Vital Signs Expression Sleeplessness (CRIES), and the Maximally Discriminate Facial Movement Coding System (MAX) [14–16].

**Neonatal Facial Coding System (NFCS)**

It is used to monitor facial actions in newborns. It was developed at the University of British Columbia, and the British Columbia children's hospital [17]. The system looks at eight indicators to measure pain intensity: brow bulge, eye squeeze, nasolabial furrow, open lips, stretched mouth (horizontal or vertical), lip purse, tout tongue, and chin quiver [17]. The indicators are recorded on videotape, coded, and scored. It has been proven reliable for short duration, acute pain in infants and neonates [18]. Since some facial actions occur in nonpainful situations, while others (horizontal and vertical mouth stretch) are present in less than 50% of painful situations, NFCS is able to discriminate between degrees of distress, but not between pain-related and nonpain-related distress [19]. The system is also difficult to assess in intubated neonates [19].

**Neonatal Infant Pain Scale (NIPS)**

It was developed at the Children's Hospital of Eastern Ontario. It is a behavioral assessment tool to measure pain [20]. The scale takes into account pain measurement before, during and after a painful procedure, scored in one-minute intervals. The indicators include: face, cry, breathing pattern, arms, legs, and state of arousal [20]. Results are obtained by summing up the scores for the six indicators (where 0 indicates no pain, and 2 indicates pain), with a maximum sore of 7 [20]. It is a good system to measure responses to acute painful stimuli. Although it has been fully validated, it is time consuming and hard to interpret in intubated infants.

**The Premature Infant Pain Profile (PIPP)**

It is a 7-indicator composite measure that was developed at the University of Toronto and McGill University to assess acute pain in preterm and term neonates. It has been validated in studies using synchronized videotaping of infants undergoing painful procedures [14, 21]. The indicators include (1) gestational age, (2) behavioral state before painful stimulus, (3) change in heart rate during stimulus, (4) change in oxygen saturation, (5) brow bulge during painful stimulus, (6) eye squeeze during stimulus, and (7) nasolabial furrow during painful stimulus [14]. Gestational age is taken into consideration. Scoring is initially done before the painful procedure. The infant is observed for 15 seconds and vital signs recorded. Infants are then observed for 30 seconds during the procedure where physiological and facial changes are recorded and scored. The score ranges from 0–21, with the higher score indicating more pain [14]. The PIPP is however burdensome and time consuming for clinical purposes, especially in the emergency department, and its use for intubated neonates remains questionable” [21].

**Crying Requires Increased Vital Signs Expression Sleeplessness (CRIES)**

It is an acronym of five physiological and behavioral variables proven to indicate neonatal pain. It is commonly used in neonates in the first month of life [15]. The scale was developed at the University of Missouri and may be recorded over time to monitor the infant's recovery or response to different interventions [22]. CRIES looks at five parameters: (1) crying, where a high pitched cry is usually associated with pain, (2) increased oxygen requirements, as neonates in pain show decrease oxygen saturation, (3) facial expression where grimacing is the expression most associated with pain, (4) vitals signs, which are usually assessed last as to not awaken or disturb the child, and (5) sleeping patterns where increased sleeplessness is associated with pain [15]. Indicators are scored from 0–2 with the maximum possible score of 10, a higher score indicating a higher pain expression [15].

**Maximally Discriminate Facial Movement Coding System (MAX)**

It is used for infants to assess emotions associated with facial expression. It looks at brow, eye, and mouth movements [16, 23]. MAX provides a system for measuring emotional signals, and identifies nine fundamental emotions: interest, joy, surprise, sadness, anger, disgust, contempt, fear, and physical distress or pain. The scoring entails 68 MAX number codes, each representing a different facial expression. The description of the expression of each number code is based on the anatomically possible movements of the facial muscles and is a description of what the face looks like when the movements have taken place [16]. Critical studies argue that MAX only includes measurements that are said to correspond with emotions and does not differentiate between anatomically distinct facial movements (inner and outer brow raise) [24, 25].

### 2.2. Toddlers

In toddlers, verbal skills remain limited and quite inconsistent. Pain-related behaviors are still the main indicator for assessments in this age group. Nonverbal behaviors, such as facial expression, limb movement, grasping, holding, and crying, are considered more reliable and objective, measures of pain than self-reports [26]. Most children of this age however are capable of voluntarily producing displays of distress, with older children displaying fewer pain behaviors (e.g., they cry, moan, and groan less often). Most two-year-old children can report the incidence and location of pain, but do not have the adequate cognitive skills to describe its severity [27]. Three-year-old children, however, can start to differentiate the severity of pain, and are able to use a three-level pain intensity scale with simple terms like “no pain, little pain or a lot” [27]. Children in this age group are usually able to participate in simple dialogue and state whether they feel pain and “how bad it is” [27]. The following section describes common scales used for this age group.

**The Children's Hospital of Eastern Ontario Pain Scales (CHEOPS)**

It is one of the earliest tools used to assess and document pain behaviors in young children [28]. It used to assess the efficacy of interventions used in alleviating pain. It includes six categories of behavior: cry, facial, child verbal, torso, touch, and legs. Each is scored separately (ranging from 0–2 or 1–3) and calculated for a pain score ranging from 4–13 [28]. Its length and changeable scoring system among categories makes it complicated and impractical to use compared to other observational scales.

**The Faces Legs Activity Cry Consolability Scale (FLACC)**

It is a behavioral scale for measuring the intensity of postprocedural pain in young children [29]. It includes five indicators (face, legs, activity, cry, and consolability) with each item ranking on a three point scale (0–2) for severity by behavioral descriptions resulting in a total score between 0–10 [29]. FLACC is an easy and practical scale to use in evaluating and measuring pain especially in pre-verbal children from 2 months to 7 years. Numerous studies have proven its validity and reliability [30].

**The COMFORT Scale**

It is a behavioral scale used to measure distress in critically ill unconscious and ventilated infants, children, and adolescents [31, 32]. This scale is composed of 8 indicators: alertness, calmness/agitation, respiratory response, physical movement, blood pressure, heart rate, muscle tone, and facial tension. Each indicator is given a score between 1 and 5 depending on behaviors displayed by the child and the total score is gathered by adding all indicators (range from 8–40). Patients are monitored for two minutes. The COMFORT scale has been proven to be clinically useful to determine if a child is adequately sedated [32].

**The Observational Scale of *Behavior*al Distress (OSBD)**

It remains the most frequently used measurement in procedure-related distress studies [33]. It consists of 11 distress behaviors identified by specialists to be associated with paediatric procedure-related distress, anxiety, and pain. Scores are calculated from summing up all 11 distress behaviors. The behaviors are usually organized into categories of growing intensity, considering their level of interference with medical procedures (e.g., moaning, flinching, and disruption of medical materials) [34]. The validity and reliability of the OSBD has been widely reported [35, 36]. Limitations of the OSBD are noted, where the explanations of the different phases of the procedure: anticipatory (when the child is waiting for the procedure), procedural (distress while the procedure is taking place), and recovery (postprocedural distress) are interchangeable among studies [35, 36]. In instances where procedural phases are constant, differences arise in initiating the procedure (e.g., venipunctures) which are frequently independent of the child's behavior, and affect the duration of the procedure and the number of observation intervals. This ultimately increases or decreases the scores [37].

**Observational Pain Scale (OPS)**

It is intended to measure pain in children aged 1 to 4 years, and is used to assess pain of short or long duration [38]. The scale was primarily produced at the University of Amsterdam in the Netherlands. The scale measures 7 parameters: facial expression, cry, breathing, torso, arms and fingers, legs and toes, and states of arousal [38]. The OPS has a simple scoring system which makes it easy to use by all healthcare professionals to obtain valid and reliable results [39]. The indicators are rated from 0-1 with a maximum score of 7, where the higher score indicates greater discomfort [38].

**The Toddler-Preschooler Postoperative Pain Scale (TPPPS)**

It is used to assess pain in young children during and after a medical or surgical procedure. It is most commonly used for children aged 1–5 years [40]. In order to observe verbal, facial, and bodily movement, the child needs to be awake. This scale relies on behavioral observations, but also includes a self report element. The TPPPS includes seven indicators divided into three pain behavior groups: vocal pain expression, (verbal complaint, cry, moan) facial pain expression (open mouth, squinted eyes, brow bulging and furrowed forehead) and bodily pain expression (restlessness, rubbing touching painful area) [41]. It is a useful tool for evaluating the effectiveness of medication administration in children, but does not measure pain intensity [42]. If a behavior is present during a 5-minute observation period, a score if 1 is given whereas a score of 0 is given if the behavior was not present. The maximum score obtained is 7, which indicates a high pain intensity [40].

### 2.3. Preschoolers

By the age of four years, most children are usually able to use 4-5 item pain discrimination scales [43]. Their ability to recognize the influence of pain appears around the age of five years when they are able to rate the intensity of pain [44]. Facial expression scales are most commonly used with this age group to obtain self-reports of pain. These scales require children to point to the face that represents how they feel or the amount of pain they are experiencing [45]. The following section describes scales commonly used with this age group.

**The Child Facial Coding System (CFCS)**

It is adapted from the neonatal facial coding system and developed for use with preschool children (aged 2–5 years). It consists of 13 facial actions: brow lower, squint, eye squeeze, blink, flared nostril, nose wrinkle, nasolabial furrow, cheek raiser, open lips, upper lip raise, lip corner puller, vertical mouth stretch, and horizontal mouth stretch [46]. The CFCS has been useful with acute short-duration procedural pain [47].

**Poker Chip Tool**

It is a tool that was developed for pre-schoolers to assess “pieces of hurt” [48]. The tool uses four poker chips, where one chip symbolizes “a little hurt” and four chips “the most hurt you could experience”. The tool is used to assess pain intensity. Health care professionals align the chips in front of the child on a flat surface, and explain, using simple terms, that the chips are “pieces of hurt”. The child is asked “how many pieces of hurt do you have right now?” [49] Although most studies focus on using it in children four to thirteen years old, adolescents have used it successfully as well [50].

**Faces Pain Scale**

It was developed by Wong and Baker and is recommended for children ages 3 and older [51]. The scale requires health care professionals to point to each face and describe the pain intensity associated with it, and then ask the child to choose the face that most accurately describes his or her pain level [51]. Most pain rating scales using faces that portray degrees of distress are divided into two categories: those starting with neutral face as the “no pain” indicator and those with a smiling face. Results showed that children exposed to smiling scale had considerably higher pain scores in the no pain categories and lower scores for positive pain than children who used the neutral faces scale [52]. A study by Chambers and colleagues indicated that children's pain ratings differ depending on the types of faces scale used, and that faces scales with smiling faces may confuse emotional states with pain ratings [52]. The revised pain scale (FPS-R) is a simplified 6 face adaption of Bieri's validated faces pain scale. It does not contain smiling faces or tears thus avoiding the confounding of affect and pain intensity [45].

**The OUCHER Scale**

It was developed by Beyer in 1980 [53]. It is an ethnically based self-report scale, which has three versions: Caucasian, African-American, and Hispanic [54, 55]. Even though it covers a wide array of patients, it still has limits. For example, females are not represented, as well as other cultures. It is used for children older than 5 years [55]. The tool has two separate scales: the numeric scale (i.e., 0–100) and the photographic scale usually used for younger children. The photographic scale entails six different pictures of one child, portraying expressions of “no hurt” to “the biggest hurt you can ever have” [56]. Children are asked to choose the picture or number that closely corresponds to the amount of pain they feel [56].

### 2.4. School-Aged Children

Health care professionals depend more comfortably on self-reports from school-aged children. Although children at this age understand pain, their use of language to report it is different from adults. At roughly 7 to 8 years of age children, begin to understand the quality of pain [57]. Self-report visual analogue and numerical scales are effective in this age group. A few pain questionnaires have also proven effective for this age such as the pediatric pain questionnaire and the adolescent pediatric pain tool [58, 59]. A brief discussion of these tools is presented here.

**Visual Analogue Scale (VAS)**

It is a horizontal line, 100 mm in length, attached to word descriptions at each end, “not hurting” or “no pain” to “hurting a whole lot” or “severe pain”. The children are asked to mark on the line the point that they feel represents their pain at this moment [60]. A color analogue scale can also be used, where darker more intense colors (i.e., red) represent more pain [61].

**Paediatric Pain Questionnaire**

It is a self-report measure to assess children and adolescents coping abilities using 8 subscales “information seeking, problem solving, seeking social support, positive self-statements, behavioral distraction, cognitive distraction, externalizing and internalizing as well as three more complex scales (approach, distraction, and emotion-focused avoidance) [58]. It contains 39 items in total, with scores ranging from 1 (“never”) to 5 (“very often”). Children or adolescents are requested to state how often they “say, do, or think” certain items when they hurt or in pain. The questionnaire usually takes about 10–15 minutes to complete [62].

**Adolescent Pediatric Pain Tool (APPT)**

It is a valid all encompassing pain assessment tool used for individual pain assessments and measures intensity, location, and quality of pain in children older than 8 years of age [63]. The APPT is most useful with children and adolescents who are experiencing complex, difficult to manage pain [59]. It consists of a body map drawing to allow children to point to the location of pain on their body and a word graphic scale to measure pain intensity. The word graphic rating scale is a 67 word list describing the different dimension of pain and a horizontal line with words attached that range from “no,” “little,” “medium,” “large,” to “worst” possible pain [59, 64–66].

### 2.5. Adolescents

Adolescents tend to minimize or deny pain, especially in front of friends, so it is important to provide them with privacy and choice. For example, they may or may not choose to have parents present. They expect developmentally appropriate information about procedures and accompanying sensations. Some adolescents regress in behavior under stress [3]. They also need to feel able to accept or refuse strategies and medications to make procedures more tolerable. To assess pain and, specifically chronic pain, the adolescent pediatric pain tool (see above section) or the McGill pain questionnaire are helpful.

**The McGill Pain Questionnaire (MPQ)**

It was developed by Melzack in 1971 [67]. It is an assessment tool that combines a list of questions about the nature and frequency of pain with a body-map diagram to pinpoint its location [68]. The questionnaire uses word lists separated into 4 classes to assess the total pain experience. The categories are (1) sensory, which contains words describing pain in terms of time, space, pressure, heat, and brightness, (2) affective category which describes pain in terms of tension, fear, and autonomic properties, (3) evaluative, and (4) miscellaneous. After the patient is done rating their pain words, the administrator allocates a numerical score, called the “Pain Rating Index” [69]. Scores vary from 0–78 with the higher score indicating greater pain [68].

## 3. Minimizing Pain during Procedures: Nonpharmacologic Methods

Pain is one of the most frequent complaints presented in paediatric emergency settings. The emergency department itself is a very stressful place for children. Thus it is important for health care providers to follow a child centered or individual approach in their assessment and management of pain and painful procedures [70]. This approach promotes the right of the child to be fully involved in the procedure, to choose, associate, and communicate. It allows freedom for children to think, experience, explore, question, and search for answers, and allows them to feel proud for doing things for themselves. It is essential to focus on the child rather than the procedure and avoid statements such as “let's just get it over with” [70]. The child and family should be active participants in the procedure. In fact, allowing parents or family members to act as positive assistants rather than negative restraints helps to reduce stress in both children and parents and minimizes the pain experience [70]. It is also essential to ensure that all procedures are truly necessary, and can be performed safely by experienced personnel. Ideally procedures should be done in a child-friendly environment, using appropriate pharmacologic and nonpharmacologic interventions with routine pain assessment and reassessment [70].

 Distraction is the most frequent intervention used in the emergency department to guide children's attention away from the painful stimuli and reduce pain and anxiety. It is most effective when adapted to the developmental level of the child [71]. Distraction techniques are often provided by nurses, parents or child life specialists. Current research has shown that distraction can lead to the reduction in procedure times, and the number of staff required for the procedure [72]. Distraction has also proven to be more economical than using certain analgesics [73]. Distraction is divided into two main categories: passive distraction, which calls for the child to remain quiet while the health care professional is actively distracting the child (i.e., by singing, talking, or reading a book) [74]. Active distraction, on the other hand, encourages the child's participation in the activities during the procedures [74]. Interventions used to minimise pain are classified into three main categories (cognitive, behavioral, or combined) [75].

**Cognitive Interventions**

They are mostly used with older children to direct attention away from procedure-related pain (e.g., counting, listening to music, non procedure-related talk) [76]. The following are a few examples of cognitive interventions:

1. *Imagery.* The child is asked to imagine an enjoyable item or experience (e.g., playing on the beach) [77].
2. *Preparation/Education/Information.* The procedure and feelings associated with the procedure are explained to child in an age appropriate manner. The child is provided with instructions about what he/she will need to do during the procedure to help them understand what to expect [78, 79].
3. *Coping statements.* The child is taught to repeat a set of positive thoughts (e.g., “I can do this” or “this will be over soon”) [80].
4. *Parental training.* The parents or family members are taught one of the above interventions to decrease their stress, as decreasing the parent's distress will often lead to a decrease in the child's distress [77].
5. *Video games and television.* These may be used to distract children from the painful procedures [81].

***Behavior*al Interventions**

They are behavioral methods to guide the child's attention away from procedure-related pain. (e.g., videotapes, games, interactive books). A few examples are:

1. *Breathing exercises.* The child is taught to concentrate on deep breathing. To engage younger children, health care professionals can use party blowers, or blowing bubbles [82].
2. *Modeling positive coping behaviors.* The child may watch another child or adult going through the procedure, and rehearse these behaviors [83].
3. *Desensitization.* This is a step-by-step approach to coping with the painful stimuli. It involves slowly introducing the procedure and tasks involved, and effectively dealing with easier tasks before moving to the next one [77].
4. *Positive reinforcement.* The child is rewarded with positive statements or concrete gifts, after the painful procedure (e.g., stickers, toys, games, small trophies) [80].
5. *Parent coaching.* The parents are instructed to enthusiastically encourage the child to use these strategies [84].

Current studies are beginning to take into consideration children's different responses to distraction interventions based on their developmental stage, maturity level, and age. Our goal in this section is to provide various forms of distraction that are proven effective with different age groups.

### 3.1. Neonates and Infants

 When performing painful procedures on infants, it is important to take into consideration the context of the procedure (i.e., is the procedure really necessary, how many painful procedures has the infant had in the past, and what was their previous pain experience) [85]. The procedural environment should also be developmentally sensitive [86]. In fact, reducing noise and lighting, use of soothing smells and clustering procedures to avoid over handling, reduces pain reactions in infants [86]. 

Distraction techniques used with this age group are mostly passive. Cognitive strategies used to reduce pain perception in infants are either visual or auditory interventions. Visual aids can include pictures, cartoons, mobile phones, and mirrors [87]. Auditory aids include music, lullabies sung by parents or health care professionals [88]. Music is more frequently being used to improve painful outcomes in infants [89]. Studies suggest that music can significantly impact behavioral reactions to pain, but not physiological measures [89]. Behavioral strategies are more common for this age group, and involve either “direct or indirect” interventions that engage the caregivers in handling the infants [90]. The combination of different strategies to provoke different senses has been shown to be more effective [91]. Examples of behavioral strategies include the following.

1. *Non-nutritive sucking,* an indirect intervention involving insertion of a pacifier or a nonlactating nipple into the infant's mouth to encourage sucking behaviors, was found to stimulate the orotactile and mechano receptors, and decrease cry durations and heart rate [92].
2. *Skin to skin contact* with the mother (kangaroo care), where the infant is positioned on the mother's exposed chest during, or after the painful procedure [93].
3. *Rocking and holding the infant, *where the infant is carried by a parent or caregiver during (if possible) and after the painful procedure and gently rocked [94].
4. *Swaddling the infant* is another similar calming technique where the infant is wrapped with its extremities close to their trunk to prevent him/her from moving around excessively [95].

### 3.2. Toddlers and Preschoolers

For young children, explaining the procedures with age appropriate information is useful, in addition to providing them with the opportunities to ask questions [70]. Examples for active distraction used with this age group include, allowing them to blow bubbles, providing toys with lots of colour or toys that light up. Initiating distracting conservations (e.g., how many brothers and sisters do you have? What did you do at your birthday party?) and deep breathing methods are also helpful for older children [74]. Passive distraction techniques include: having the parents or child life specialist read age appropriate books, sing songs, and practicing “blowing out birthday candles” with the child [74].

### 3.3. School-Aged Children

Older children have a better understanding of procedures and why they are being done, thus providing them with age appropriate information is also important [70]. Providing children with a choice (e.g., sit or lie down, choose which hand) helps them feel in control of the situation [70]. Asking parents about their child's previous pain experiences and coping mechanisms helps health care professionals identify appropriate interventions to use with the child. Educating school-aged children about passive and active techniques available will help them cope with the distress and anxiety of the procedure [70]. Active techniques for this age group include blowing bubbles, singing songs, squeeze balls, relaxation breathing and playing with electronic devices [74]. Passive distraction can include watching videos, listening to music on headphones, reading a book to the child or telling them a story [74].

### 3.4. Adolescents

It is essential to always ensure a private setting for procedures with adolescents especially as they sometimes tend to deny pain in front of friends, and family. Giving them the power to choose the type of distraction, or whether they want friends and family present is helpful [70]. Striking conversations, using squeeze balls or having them play with electronic devices are examples of active techniques, while passive distractions include watching videos, training them to breathe deeply (in from the nose, count to 5 and out through the mouth), and listening to music [74].

## 4. Conclusion

Although there is an overwhelming amount of data regarding effective paediatric pain assessment and management, it is often not being effectively applied. Current studies demonstrate pain management in children remains undertreated. It is the responsibility of health care professionals to educate their peers and advocate for appropriate pain treatment in children. Infants and children present a unique challenge that necessitate consideration of their age, developmental level, cognitive and communication skills, previous pain experiences, and associated beliefs. There is a need for more research to illuminate optimal pain management and strategies that take these special needs into consideration, to improve the treatment of pain in children.

## Figures and Tables

**Table 1:** Pain assessment scales.

Ages	Scales	Description/indicators	Websites and references	Validity
Preterm to full-term infants	Premature Infant Pain Profile (PIPP)	Gestational age, behavioral state before painful stimulus, change in heart rate during stimulus, change in oxygen saturation, brow bulge, eye squeeze nasolabial furrow	http://www.cebp.nl/vault_public/filesystem/?ID=1467	Stevens et al., [14, 86]

Preterm to full-term infants	Neonatal Facial Coding System (NFCS)	Brow bulge, eye squeeze, nasolabial furrow, open lips, stretched mouth (horizontal or vertical), lip purse, tout tongue, and chin quiver	http://www.cebp.nl/vault_public/filesystem/?ID=1425	Grunau et al., [18]

Preterm to full-term infants	Neonatal Infant Pain scale (NIPS)	Face, cry, breathing pattern, arms, legs, and state of arousal	http://www.cebp.nl/vault_public/filesystem/?ID=1426	Lawrence et al., [20]

32–60 weeks	Crying Requires Increased vital signs Expression Sleeplessness (CRIES)	Crying, increased oxygen requirements, expression, vitals signs, sleeping	http://www.cebp.nl/vault_public/filesystem/?ID=1295	Krechel and Bildner, [15]

Infants	Maximally discriminate facial movement coding system (MAX)	Brow, eye, and mouth movement	Izard C. Maximally Discriminate Facial Coding System,1983	Izard [23]

1–7 years	Chidren's Hospital of Eastern Ontario Pain Scale (CHEOPS)	Cry, facial, child verbal, torso, touch, legs	http://www.cebp.nl/vault_public/filesystem/?ID=1274.	McGrath et al., [28]

Infancy to 7 years	The Faces Legs Activity Cry Consolability Scale (FLACC)	Face, legs, activity, cry, and consolability	http://www2.massgeneral.org/painrelief/pcs_pain_files/app_d_flacc.pdf.	Merkel et al., [29]

1–4 years	Observational Pain Scale	Facial expression, cry, breathing, torso, arms and fingers, legs and toes, and states of arousal	https://www.cebp.nl/vault_public/filesystem/?ID=1451.	Boelen-Ven der Loo et al., [38]

1–5 years	Toddler-Preschooler Postoperative Pain Scale (TPPPS)	Vocal pain expression, facial pain expression, bodily pain expression	http://www.cebp.nl/vault_public/filesystem/?ID=1490.	Tarbell et al., [40]

1–6 years	Child Facial Coding System (CFCS)	Facial actions: brow lower, squint, eye squeeze, blink, flared nostril, nose wrinkle, nasolabial furrow, cheek raiser, open lips, upper lip raise, lip corner puller, vertical mouth stretch, and horizontal mouth stretch.	Manual: Pediatric Pain-Science Helping Children, IWK Grace Health Centre, Dalhousie University & the University of British Columbia C.T. (copyright 1996)	Gilbert et al., [47]

3–7 years	COMFORT Scale	Calmness/agitation, respiratory response, physical movement, blood pressure, heart rate, muscle tone, and facial tension	http://painconsortium.nih.gov/pain_scales/COMFORT_Scale.pdf	Ambuel 1990 [31]

≥3 years	Faces Pain Scale	Pain intensity Faces correspond to pain intensity	http://painconsortium.nih.gov/pain_scles/index.html, http://www.usask.ca/childpain/fpsr/	Wong and Baker, [51], Hicks et al., [45]

3–13 years	The Observational Scale of behavioral Distress (OSBD)	Eleven behaviors related to pain and/or anxiety		Elliot 1987 [34], Tucker et al., [35]

4–13 years	Poker Chip Tool	Pain intensity Poker chips represent “pieces of pain”	http://www.painresearch.utah.edu/cancerpain/attachb7.html	Hester et al., [49]

≥5 years	Oucher Scale	Pain intensityFaces correspond to pain intensity	http://www.oucher.org/the_scales.html	Beyer et al., [56]

7 years	Visual Analogue Scale (VAS)	Pain intensity (numeric, color)	http://www.cebp.nl/vault_public/filesystem/?ID=1478.	

8–17 years	Pediatric Pain Questionnaire	Information seeking, problem solving, seeking social support, positive self-statements, behavioral distraction, cognitive distraction, externalizing, internalizing/	http://www.seattlechildrens.org/pdf/pediatric_pain_questionnaire.pdf	Varni et al., [58]

8–17 years	Adolescent Pediatric Pain Tool (APPT)	Intensity, location, and quality of pain		Savedra et al., [59]

≥12 years	McGill Pain Questionnaire	Sensory and affective pain experience	http://www.cebp.nl/vault_public/filesystem/?ID=1400	Melzack, [68]
